# Endoscopic Management of Upper Tract Urothelial Carcinoma

**DOI:** 10.1155/2009/620604

**Published:** 2009-01-04

**Authors:** K. Moore, J. Khastgir, M. Ghei

**Affiliations:** Department of Urology, Morriston Hospital, Swansea SA6 6NL, UK

## Abstract

Nephroureterectomy is currently the gold standard for management of upper urinary tract urothelial carcinoma despite it results. This review article in the loss of a renal unit. The ultimate aim of endoscopic management of this condition is cancer control whilst preserving renal function and the integrity of the urinary tract. Endoscopic treatments of upper tract TCC include the antegrade percutaneous and retrograde ureteroscopic approaches. This review article summarizes the endoscopic management of upper tract urothelial carcinoma, surveillance of the disease after endoscopic management and adjuvant therapy. The main message regarding endoscopic management of upper tract urothelial cancer is that patients must be carefully selected. Patient selection is based on tumour size, grade, and multifocality. Single low-grade tumours, less than 1.5 cm in size, generally have a good outcome with endoscopic treatment provided that they have regular ureteroscopic surveillance. Ureteroscopic treatment of high-grade tumours is essentially palliative. It is essential that patients are motivated and compliant as lifetime follow-up is necessary. However, until large randomized trials with long-term follow-up are performed, endoscopic management cannot be considered a standard treatment and should be limited to poor performance status patients.

## 1. Introduction

Primary urothelial carcinoma of the upper urinary tract accounts for 5% of all urothelial tumours and 7–10% of all kidney tumours. Nephroureterectomy including a cuff of bladder has been the “gold standard” treatment despite the associated morbidity and loss of a renal unit. Currently, laparoscopic approaches have reduced the size of the incision, length of hospital stay, postoperative pain, and morbidity and give oncological outcomes similar to those of open surgery. However, a laparoscopic nephroureterectomy is still radical surgery that does not spare the renal parenchyma.

The ultimate aim of endoscopic management is cancer control whilst preserving renal function and the integrity of the urinary tract. These procedures were initially reserved for patients with solitary kidneys, bilateral disease, or renal insufficiency but are starting to gain acceptance in the management of small, low-stage, low-grade tumours in patients with normal contralateral kidneys. Endoscopic treatment of the upper urinary tract includes antegrade percutaneous and retrograde ureteroscopic approaches which were first described in the mid 1980s.

## 2. Tumour Grade and Stage

A key point in choosing the optimum treatment for upper tract urothelial carcinoma is correct staging by endoscopic evaluation and biopsy. There are significant differences in 5 year survival rates ranging from 60–90% in Ta/1 and CIS disease to only 5% in T3/4 disease. The most important factors for survival are tumour stage, grade, and multifocality [1].

In a study of 130 consecutive nephroureterectomy specimens, tumour stage significantly correlated with tumour grade. Five percent of patients with low-grade UTUC had pathologic stage pT2 or higher, while 65% of patients with high-grade tumours had pathologic stage pT2 or higher [2]. Similarly, Murphy et al. reported that 47 of 49 patients (96%) with grade 1 upper tract transitional cell carcinoma also had stage 1 disease [3].

Tumour grade is also related to recurrence rate with Zincke reporting that only 5% of 21 patients with grade 1 or 2 developed a recurrence compared with 50% with grade 3 or 4 disease [4]. Orihela reported that in 14 patients recurrences were almost exclusively in those with multifocal, high-grade, invasive tumours [5]. In general, recurrences are unlikely in patients with single, low grade small tumours confined to the mucosa with no history of concurrent urothelial tumours elsewhere in the urinary tract. Upper tract recurrence rates were 28.5% in a group with a history of bladder lesions compared to 16.6% in a group without bladder disease [6].

However, diagnosis of ureteric lesions is not straight forward. In a study by El-Hakim et al., ureteroscopic appearances of upper tract urothelial carcinoma was only 70% accurate in determining the grade and they suggested biopsies must be taken to in order to determine the true grade [7]. In contrast, a study by Keeley et al. showed transitional cell carcinoma grade on ureteroscopy accurately predicted tumour grade and stage in the surgical specimens. They observed that, of the 30 low or moderate grade ureteroscopic specimens, 27 (90%) proved to be low or moderate grade transitional cell carcinoma in the surgical specimens, while 11 of 12 high grade ureteroscopic specimens (91.6%) proved to be high grade transitional cell carcinoma (*P* < .0001). In 30 low or moderate grade ureteroscopic specimens, 26 (86.6%) had a low stage (Ta or T1) tumour. In contrast, 8 of 12 high grade ureteroscopic specimens (66.7%) had invasive tumour (stage T2 or T3) in the surgical specimen (*P* = .0006) [8]. These same authors also noted how crucial the techniques for handling and processing the small samples obtained via ureteroscopy are. They found that sending multiple samples, including saline washes before and after biopsy, improved the ability to grade tumours ureteroscopically from 42.9% to 90% [8] .

A further study by Williams et al. to determine the accuracy of ureteroscopic biopsy in predicting the histopathology of upper tract TCC looked at 30 biopsies taken between 1998 and 2006. At nephroureterectomy, 2 cases were found to have no tumour. Of the remaining 28 cases, the biopsy grade proved to be identical in 21 (75%). 17 of 25 (68%) of grade 1-2 ureteroscopic specimens had a low stage (T0, Ta, or T1) tumour. In contrast, 3 of 5 (60%) high grade specimens had invasive tumour (T2 or T3). They concluded that ureteroscopic inspection and biopsy provided accurate information regarding the grade and stage of upper tract TCC [9].

Tumour size also appears to influence recurrence rate. One study reported that only 7 of 19 renal units (36%) with tumours larger than 1.5 cm were ever rendered tumour free and 3 of 6 tumour free renal units subsequently developed a recurrence. In contrast, 20 of 22 (91%) of renal units with initial tumours less than 1.5 cm were rendered tumour free and only 5 (25%) tumour free kidneys had recurrences [10]. This finding was echoed by Johnson et al. who found that patients presenting with tumours >1.5 cm in size had a higher incidence of recurrence and recurred earlier [11]. Johnson et al. confirmed the aggressive nature of high grade disease. In a cohort of 63 patients, tumour progression was seen in 83% of patients with high grade ureteric urothelial carcinoma when nephroureterectomy was not performed [11].

## 3. Ureteroscopic Management

Developments in ureteroscopic instruments and techniques now allow for ureteroscopic access to the entire upper urinary tract. Small diameter rigid and flexible ureteroscopies with greater deflecting abilities have been combined with endoscopic biopsy techniques and devices for tissue ablation to offer practical approaches to upper urinary tract tumours. Particularly, the holmium:YAG and neodymium:YAG lasers used to cauterize and ablate tumours, delivered through small-diameter, flexible fibres, have allowed treatment of relatively large tumours whilst maintaining homeostasis [11]. Electrosurgical techniques were first used for the treatment of ureteric neoplasms. They are used in a similar way to resectoscopes for other procedures but because of the rigid design of the resectoscope, its use is primarily confined to the distal ureter. Given the thin wall of the ureter, care should be taken to avoid resecting through the full thickness of the wall and also to avoid fulgurating a large area of the ureter as this increases the chances of subsequent stricture formation. Simple fulguration with an electrocautery probe is another electrosurgical technique suitable for very small lesions or for the base of the tumour after removal of the bulk of the lesion [12].

The neodymium:YAG laser has been used widely for the treatment of both bladder and upper tract tumours. The fibre is directed at and placed in close approximation to the tumour, activated at 20 to 30 w and moved over the surface to coagulate the tissue. The laser penetrates to a depth of 5-6 mm. The coagulated tissue is removed with graspers to expose further portions of the tumour which can be treated in the same fashion.

The holmium:YAG laser both coagulates and ablates tissue penetrating to a depth of 0.5 mm. This is useful for ureteric lesions as it can ablate and remove an occlusive tumour opening up the lumen. Irrigation is needed to clear the visual field of tissue debris during treatment.

The two lasers can be used in combination. The neodymium:YAG laser, penetrating to a depth of several millimetres, is used to coagulate the major volume of the tumour, then the coagulated tissue can be removed with the holmium:YAG laser [12].

Schmeller compared laser ablation with electrocautery and found fewer strictures developed in the laser group [13]. However, Martinez-Pineiro et al. found that their laser results did not offer significant benefit compared with electrocautery [6]. Data on electrocautery versus laser treatment is scant (probably due to the small cohort of patients being treated in this manner) but to date there is no convincing evidence that the efficacy of tumour destruction is affected by the method used.

All ureteroscopic interventions should be followed with short term ureteral stenting to prevent any postoperative obstructive sequelae [12].

Complications of ureteroscopic management occur in 8–13% and are mostly minor including perforation in 1-4% (managed by ureteric stenting or percutaneous drainage) and ureteric strictures in 4.9–13.6%. Most strictures can be managed by stenting, laser incision, or balloon dilatation [14].

In a study by Keeley et al., between 1985 and 1995, 92 patients were diagnosed with upper tract TCC. 46 had a diagnostic ureteroscopy followed by open extirpation and 46 had some form of endoscopic treatment. 8/46 had open surgery following endoscopic therapy and 38 (41 kidneys) had ureteroscopic treatment and follow-up. Semirigid and flexible ureteroscopes were used to examine the collecting system, tumours were biopsied then treated with fulguration, the neodymium:YAG laser and/or the holmium:YAG laser. Patients were treated every 6 to 12 weeks until tumour free and then followed up with further ureteroscopy. At least 1 follow-up ureteroscopic examination was performed in all 38 patients. Of the 41 renal units, 28 (68%) were rendered tumour free after an average of 1.57 ureteroscopic treatments. Complications were generally related to comorbid disease, 1 patient with a solitary kidney developed an episode of acute renal failure with clot retention but recovered to baseline renal function. No patient required a blood transfusion or emergency open surgery for bleeding. 2 patients had ureteric strictures, 1 with a history of pelvic radiotherapy for bladder cancer and 1 following neodymium:YAG laser treatment of a proximal ureteric tumour. No ureteric perforations were noted [10].

Chen and Bagley followed 23 patients with a normal contralateral kidney for a mean of 35 months after initial ureteroscopic treatment of upper tract transitional cell carcinoma (range 8 to 103). 22 tumours were grade 1 to 2 and 1 was grade 2 to 3. There were multiple recurrences (treated ureteroscopically) in 15 of 23 patients (65%) and no recurrences in 8 (35%). Average time to recurrence was 9.5 months (range 2 to 53) with an average of 4 recurrences (1 to 14). There were no metastases or mortality from transitional cell carcinoma. At completion of the study, 4 patients (17%) had persistent disease and 4 (17%) elected to undergo nephroureterectomy. Complications included ureteral strictures in 2 patients treated for distal ureteral tumours. The strictures were treated with endoscopic dilation [15].

A study from Madrid reported a failure of ureteroscopy in 11 of 39 patients (28.2%), mainly due to inability to reach pelvic tumours or to destroy the tumour. Four of these patients were successfully treated by a percutaneous approach and 7 required nephroureterectomy [6]. Similarly, Blute et al. reported a high ureteroscopic failure rate in 14/22 (63.6%) of patients with renal pelvic tumours, indicating that ureteroscopy is not the best procedure for most tumours of the renal pelvis and that these neoplasms are best managed percutaneously [16].

## 4. Percutaneous Nephroscopic Management

Although ureteroscopy has the theoretical benefit of preserving a closed urinary system, percutaneous access may be necessary when tumours are not accessible via a retrograde route or for larger tumours. Percutaneous nephroscopy offers better visualisation of the renal pelvis while accommodating larger calibre instruments capable of handling a larger tumour burden [12].

After establishing a percutaneous tract, the lesion is initially biopsied and then bebulked. As there is a larger access tract, cold cup biopsy forceps can be used through a standard nephroscope or a cutting loop from a resectoscope. The base of the lesion is resected and sent for histological evaluation and haemostasis is achieved by electrocauterey or laser ablation as previously described. The established nephrostomy tract can be maintained, allowing for repeated treatment or administration of topical adjuvant therapy [12].

Complication rates with the percutaneous approach are low and include blood transfusion in <20% and less commonly, PUJ obstruction from stricture, adjacent organ injury, and pleural injury [1]. Tumour seeding along the nephrostomy tract has been reported [17]. Larger series though, failed to find tract recurrences confirming that this phenomenon is rare. Precautions suggested to minimize seeding include use of an Amplatz sheath to decrease intrarenal pressure during manipulations and immediate irrigation of the collecting system and percutaneous tract with a 5-florouracil solution. One author suggested placing a radioactive iridium wire in the percutaneous tract [18].

Goel et al. reported on 5 year outcomes of 24 patients who underwent primary percutaneous resection of the urothelial tumour. Patients with low stage pT0-1 disease were treated primarily with percutaneous surgery. Patients with multi-segmental pelvicaliceal system involvement, stage greater than pT1, high grade histology or additional ureteral tumours were considered for nephroureterectomy. Topical chemotherapy (mitomycin C or epirubicin) was administered via nephrostomy tube or intravesical instillation after Double-J stent insertion. Surveillance included upper tract cytology, nephroscopy or fiberoptic ureterorenoscopy.

Of the 24, 2 cases had squamous cell carcinoma, 5 had grade 3 transitional cell carcinoma, 15 had grade 1 to 2 transitional cell carcinoma and 2 had no tumour. Control was established with initial percutaneous resection in 18 (75%) cases and second look nephroscopy in 4. All patients with high-grade disease died of malignancy except one (with no further treatment) and 6 of the 15 patients with low-grade noninvasive transitional cell carcinoma underwent nephroureterectomy during follow-up either due to progression of disease, concomitant tumour, or complications. Two patients with solitary kidneys died of renal failure unrelated to malignancy. High grade tumours or tumours greater than T1 were treated with nephroureterectomy early during management. There was no perioperative mortality and in 9 (60%) of the low-grade cases the kidneys were preserved at mean follow-up [19]. 

In a more recent study, Palou et al. retrospectively reviewed 34 patients who had percutaneous management of their upper tract TCC. 15% had grade 3 tumours with either a solitary kidney or bilateral disease. During a 4.25-year follow-up, recurrence was found in 44% at a median time of 24 months. 9 cases required nephroureterectomy. Renal preservation was achieved in 74%. Overall survival and cancer specific survival was 71% and 93%, respectively [20].

Rouprêt et al. reported on the results of 24 patients who underwent a percutaneous approach to their tumour. Median follow-up was 62 months with recurrences detected in 8/24 at a median time of 17 months. 3 recurrences were in the ipsilateral ureter, 1 in the contralateral ureter, and 4 in the bladder. Five patients with high grade and/or invasive tumour subsequently underwent an open nephroureterectomy, one immediately and the others during follow-up. 5/24 (20.8%) of the patients have died, and 4 of these deaths were attributed to disease progression. They reported 5-year disease specific survival rates as 79.5% and tumour free survival rates as 68%. 4 patients developed perioperative complications; 3 required blood transfusion and 1 developed a collection (which was managed with antibiotics) after inadvertent puncture of the bowel [21].

## 5. Surveillance

Unlike traditional management with nephroureterectomy, endoscopic treatment of upper tract TCC requires strict ureteroscopic surveillance as both the ureteroscopic and percutaneous approach are associated with a high risk of ipsilateral recurrence. Endoscopic follow-up has been shown to be more sensitive than radiological examination as IVU may miss up to 75% of small recurrences [9]. Surveillance ureteroscopy is usually performed at 3 and 6 months, then 6 monthly for a year, then annually, and it requires a counselled, well-motivated patients to strictly adhere to the follow-up protocol. Surveillance needs to be performed for an indefinite time interval as recurrences have been reported after 8 years of follow-up [22].

In an effort to reduce the anaesthetic risks, costs and time of surveillance, a study from New York reported on their 16 year experience of office-based ureteroscopy for surveillance of TCC after initial endoscopic ablation. 10 patients were treated with endoscopic ablation for TCC. A total of 67 (range 1 to 19 per patient) surveillance ureteroscopies were performed in the office setting. This procedure was performed without anaesthetic (only lignocaine jelly to the urethra) using a flexible ureteroscope. This revealed 7 upper tract TCC recurrences in 5 patients. A thorough ureteroscopic examination in the operating room revealed that only one patient had more extensive disease than was indicated in the office based ureteroscopy. All patients tolerated the office based procedure well with minimal discomfort. There were no acute complications [23].

Urinalysis with dipstick and microscopic examination is an attractive surveillance tool as it is noninvasive, inexpensive, and can be performed in an outpatient setting giving an immediate result. In patients with recurrent upper tract transitional cell carcinoma, urinalysis had a low sensitivity (36.3%) but high specificity (90.6%) in detecting recurrent disease. This low detection rate may be due to the low grade nature of most upper tract transitional cell carcinomas managed with local resection as low grade tumours are less likely to shed diagnostic cells [24]. In a series by Xia, voided cytologies were positive in 33% of grade 1, 71% of grade 2 and 100% of grade 3 upper tract tumours. In recent years adjunct diagnostic techniques such as immunocytologic staining or fluorescence in situ hybridisation have been used for evaluating the presence or absence of malignant cells in urine [25].

## 6. Adjuvant Therapy

A substantial proportion of patients with endoscopically managed upper tract urothelial carcinoma will develop a recurrence. Adjuvant topical immunotherapy or chemotherapies have been used in an attempt to reduce the risk of tumour recurrence. The most commonly instilled agents are mitomycin-C and BCG. Method of instillation, depending on the approach to the tumour, can be performed via a retrograde ureteral catheter or through a percutaneous nephrostomy tube. Most published reports involve few patients with short follow-up and a relatively high complication rate.

Orihuela and Smith found a lower recurrence rate (16.6% versus 80%) in those patients treated with BCG compared to those who did not recieve adjuvant treatment but a further study by the same group showed no survival advantage [5]. Sharpe et al. reported on the use of BCG via retrograde ureteral catheters in 17 kidneys of 11 patients with abnormal cytology. At a mean follow-up of 36 months cytology had normalised in 8 of 11 patients. 1 patient developed a fever and was treated with antituberculous drugs. In a further study of 18 patients treated with BCG, 7 developed fever on 14 occasions and 1 patient died of sepsis despite prophylactic IV antibiotics. In terms of efficacy, no significant difference was found between the patients treated with BCG and those who were not [26].

Keele et al. looked at 19 patients who underwent a total of 28 treatments with mitomycin C after ureteroscopic treatment for high volume, recurrent or multifocal disease. Following 1 to 4 treatments with MMC, 11 of 19 (58%) were rendered free of disease, 4 required nephroureterectomy for persistent or recurrent disease and no patients developed local or distant progression of disease or any significant side effects [26]. In a study that reported on 14 patients who received MMC, one patient died of aplastic anaemia and sepsis secondary to extravasation during treatment. This same study also found a lower rate of recurrence among patients treated with MMC or BCG compared to those treated with thiotepa or oral 5-fluorouracil [6].

As yet, no study has shown statistical improvement regarding survival and recurrence rates and no protocol of treatment has been accepted. Randomized multicenter trials are needed to assess the efficacy of adjuvant agents.

## 7. Endoscopic Treatment versus Radical Nephroureterectomy

Rouprêt et al. compared the outcomes in patients who had undergone either open nephroureterectomy or endoscopic surgery (ureteroscopic or percutaneous management) for upper urinary tract transitional cell carcinoma. A retrospective review of the data for patients treated surgically for upper urinary tract transitional cell carcinoma from 1990 to 2004 was performed. Data were analyzed for 97 patients. The surgical procedure was open nephroureterectomy in 54 patients, ureteroscopy in 27, and percutaneous endoscopic ablation in 16. In patients with low-grade tumours (*n* = 46), the 5-year disease-specific survival rate after nephroureterectomy, ureteroscopy, and percutaneous endoscopy was 84%, 80.7%, and 80%, respectively (*P* = .89); the corresponding 5-year tumour-free survival rates were 75.3%, 71.5%, and 72% (*P* = .78) [27].

Lee et al. had similar findings when reviewing their 13 year experience of percuaneous management of upper tract urothelial carcinoma. They found no significant difference in overall survival when compared with nephroureterctomy. Regardless of treatment modality, patients with low grade lesions did well where as those with high grade lesions were predisposed to tumour recurrence and progression. Also, recurrence rates of bladder TCC appear to be similar after radical nephrectomy or endoscopic surgery [28].

Boorjian et al. reviewed 121 patients who underwent a nephroureterectomy for upper tract TCC over a 10-year period. In comparing patients who underwent nephroureterectomy on the basis of positive cytology findings and filling defects on contrast imaging (*n* = 34) with patients who had nephroureterectomy after ureteroscopic biopsy (*n* = 75) and patients who had nephroureterectomy after ureteroscopic biopsy and laser ablation (*n* = 12), they found no significant difference in postoperative disease status. Disease free rates in the 3 groups were 85.3%, 81.3%, and 83.3%, respectively [29].

## 8. Conclusions

Many reports of endoscopic surgery for upper tract urothelial carcinoma have emerged but only a few have a reasonable number of patients. Most series are small, with all types of indications (elective and palliative) and tumour characteristics (grade, stage, size, location).

The main message from series of endoscopic management of upper tract urothelial cancer is that patients must be carefully selected. Patient selection is based on tumour size, grade, and multifocality. Single low-grade tumours, less than 1.5 cm in size generally have a good outcome with endoscopic treatment provided they have regular ureteroscopic surveillance. Ureteroscopic treatment of high-grade tumours is essentially palliative. It is essential that patients are motivated and compliant as lifetime follow-up is necessary.

Recurrence rates are high but these recurrences can be treated with further endourological therapy or radical surgery as studies have shown that endological manipulation does not have a negative impact on survival.

The endoscopic approach can be mooted as an alternative approach to nephroureterectomy in poor performance status patients, but, until large, randomized trials with long-term follow-up are performed, it cannot be considered as a standard treatment.
